# Increased serum prolactin level may indicate more migraine attack frequency

**DOI:** 10.1002/brb3.3063

**Published:** 2023-05-15

**Authors:** Mansoureh Togha, Shiva Nematgorgani, Zeinab Ghorbani, Pegah Rafiee, Samaneh Haghighi

**Affiliations:** ^1^ Headache Department, Iranian Centre of Neurological Research, Neuroscience Institute Tehran University of Medical Sciences Tehran Iran; ^2^ Headache Department, Neurology Ward, Sina Hospital, School of Medicine Tehran University of Medical Sciences Tehran Iran; ^3^ Cardiovascular Diseases Research Center, Department of Cardiology, Heshmat Hospital, School of Medicine Guilan University of Medical Sciences Rasht Iran; ^4^ Department of Clinical Nutrition School of Medicine Guilan University of Medical Sciences Rasht Iran

**Keywords:** headache, migraine, Prolactin

## Abstract

**Objectives:**

Migraine is a common, multifactorial disorder. The exact pathomechanism of migraine remains unclear. Studies have revealed changes in serum prolactin (PRL) levels in relation to migraine, although the results have been inconsistent. The present case‐control study assessed the serum level of prolactin in migraine patients.

**Materials and methods:**

In this case‐control study, participants were divided into chronic migraine (CM; *n* = 39), episodic migraine in ictal (during an attack), and interictal (between attacks) phases (*n* = 63, *n* = 37, respectively) along with 30 age‐ and sex‐matched headache‐free controls. After obtaining demographic, anthropometric data, and headache characteristics, blood samples were gathered and analyzed to evaluate the serum levels of prolactin (ng/mL).

**Results:**

A significant difference was observed between the control, CM, and ictal EM, and interictal EM groups. The mean ± SD serum prolactin levels of the chronic migraineurs (1.82 ± 0.94) and those with ictal EM (1.93 ± 1.70) were comparable and were significantly higher than for interictal EM patients (0.82 ± 0.46) and the headache‐free control subjects (0.49 ± 0.15; *p* < .001). Although the mean serum concentration of prolactin for the interictal EM group tended to be higher than for control individuals, this difference was not statistically significant. The Spearman's correlation test also showed significant correlations between the serum prolactin levels and the number of headaches days among migraineurs.

**Conclusion:**

The findings suggest that there might be an association between increased prolactin concentrations and migraine headache induction and progression. Further detailed and well‐designed studies are needed to confirm the importance of serum prolactin levels in the pathogenesis of migraine headaches.

## Graphical abstract

The present case‐control study assessed the serum level of prolactin in migraine patients. The findings suggest that changes in prolactin levels are related to the pathogenesis of migraine attacks and may contribute to the progression of migraine headaches.

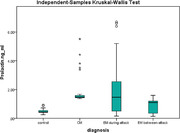

## 1 INTRODUCTION

Migraine, as characterized by moderate to severe, pulsatile, recurrent, unilateral, and 4–72 h headache attacks (Headache Classification Committee of the International Headache Society [IHS], 2018). Its prevalence peaks in those aged 35−39 years (Ashina et al., 2021). Extreme sensitivity to light, sound and smell along with vomiting and nausea are typical migraine symptoms (Edvinsson et al., 2018). Migraine is the most prevalent medical disorder and the second leading cause of disability globally (Ashina et al., 2021).

Migraine patients can be classified as either chronic migraine (CM) or episodic migraine (EM). An EM is characterized as fewer than 15 migraine headaches per month. CM is characterized as more than 15 headache days per month over the course of 3 months, of which at least eight days have migraine characteristics or show response to triptans (Headache Classification Committee of the International Headache Society [IHS], 2018). On average, migraine occurs in 15% of the population in 1 year (Ashina et al., 2021) with a marked sex difference. Migraine is two to three times more prevalent in females than in males (Tire et al., 2019; Woldeamanuel & Cowan, 2017).

Several factors cause sex differences in migraine, one of which is sex hormones such as estrogen, progesterone, and prolactin. There is increasing evidence that a higher level of prolactin in women can promote pain. In addition, it may be because the expression of a prolactin receptor in female nociceptors and their responses to external stimuli are different (Gazerani, 2021). Although the exact pathophysiology of migraine headache is unknown, it has been shown that it appears to occur in relation to a mixture of environmental and genetic factors (Goadsby et al., 2017). It is likely that migraine attacks are caused by both peripheral and central mechanisms. It remains unclear whether migraine attacks begin in the peripheral or central nervous system (Do et al., 2023). Genetic changes in ion channels, the trigeminal release of CGRP from C‐fibers, and cranial vasodilatation in the central nervous system (CNS) may have roles in initiating migraine attacks (Do et al., 2023; Edvinsson et al., 2012). Several events in the trigeminal ganglion can give rise to migraine attacks, including neuronal hyperexcitability in the cortical region, cortical spreading depression (CSD) leading to the activation and sensitization of trigeminal nociceptors, but is still controversial, and cranial vasodilatation and the release of inflammatory molecules in the meninges (Artero‐Morales & González‐Rodríguez, 2018; Charles, 2018; Zhang et al., 2010).

Migraine attack can be triggered by several factors that include stress, changes in sleep patterns, specific sensory stimuli (light, sound, and smell), alcohol, foods, and hormonal fluctuations (Gazerani & Cairns, 2018). Hormones that act on the pituitary‐hypothalamic axis are thought to be important in the pathophysiology of migraine. Of this axis, prolactin, a polypeptide composed of 199 amino acids, is secreted from the lactotrophic cells of the anterior pituitary gland (Saleem et al., 2018). Prolactin secretion is controlled by dopamine, which is a inhibitory regulator (Saleem et al., 2018) and is positively regulated by stress, exercise, BMI, circadian rhythms, serotonin and proinflammatory cytokines such as TNF‐α and IL‐6 (Corona et al., 2011; Fojtíková et al., 2010; Roelfsema et al., 2012).

Studies have shown that there is an association between increased prolactin levels and blood pressure, cardiovascular disease, diabetes type 2 (Corona et al., 2011), rheumatoid arthritis (Fojtíková et al., 2010), inflammatory diseases, pain (Patil et al., 2013), and neurological diseases such as headache (Pineyro et al., 2017) and multiple sclerosis (Wei et al., 2017). It is interesting to note that the level of prolactin has been associated with some CNS manifestations and disease activity.

Reports about the level of prolactin in migraineurs are conflicted. Some studies have reported that serum prolactin levels seem to be higher in migraineurs than in headache‐free subjects (Chen et al., 2020; Guldiken et al., 2011; Noori‐Zadeh et al., 2020). Other studies have reported contrary results (Masoud & Fakharian, 2005; Murialdo et al., 1986). Furthermore, most studies have been based on small sample sizes and did not examine different categories of migraine (Alia et al., 2013; Bosco et al., 2008; Masoud & Fakharian, 2005). The evidence suggests that it is necessary to determine the exact role of prolactin in the pathogenesis of migraine with the use of well‐designed studies having sufficient sample sizes. To this end, we designed a study to assess serum prolactin levels in subjects with EM and CM and compared these levels with an age‐ and sex‐matched headache‐free control group to investigate the relationship between prolactin and migraine.

## 2 MATERIALS AND METHODS

### 2.1 Study population

This case‐control study examined chronic migraine (CM; *n* = 39), episodic migraine in ictal (during an attack) and interictal (between attacks) phases (*n* = 63, *n* = 37, respectively) and 30 headache‐free age‐ and sex‐matched control subjects. Recruitment was from September 2017 to June 2020. The migraine groups were recruited from the Sina University Hospital Headache Clinic (a tertiary headache clinic) and the control group was selected from age‐ and sex‐matched headache‐free candidates from the general population (hospital staff and patient companions). Individuals in both groups were chosen from respondents to an advertisement (the posters were mostly displayed in headache clinics and their immediate surroundings, but they were also displayed throughout the hospital) for voluntary participation in a case‐control study on the serum level of prolactin in migraine.

A neurologist who is an expert headache specialist diagnosed the migraines after examination according to the International Headache Society Criteria (ICHD‐III) (Headache Classification Committee of the International Headache Society [IHS], 2018). Subjects who had EM or CM for at least 6 months before enrollment were selected. The inclusion criteria for enrollment in the present study were as follows: being 18 to 65 years of age; BMI of 18.5 to 35 kg/m^2^; not being diagnosed with medication overuse headache (MOH); having no medical history of inflammatory, infectious, allergic or immune disorders; having no history of cardiovascular or endocrinological diseases, liver or kidney disorders, or other neurological or chronic diseases, such as epilepsy, Parkinson's disease, multiple sclerosis, or Alzheimer's disease. Subjects were excluded if they were pregnant, or breastfeeding.

Subjects were excluded from the study if they had a history of any of the disorders listed in the inclusion criteria or who were not willing to sign the consent form. The study protocol complied with the guidelines of the 2013 version of the Helsinki Declaration. The study protocol was approved by the National Institute for Medical Research Development (NIMAD; grant no. 957537) and was confirmed by the ethics committee of NIMAD (ID: IR.NIMAD.REC.1396.054).

### 2.2 Headache diaries and visual analog scale

At the beginning of the study, the required information about demographic and anthropometric characteristics, medications, and medical history were collected from the participants. After that, blood samples were collected from the control subjects. The migraine subjects were diagnosed according to ICHD3 criteria by the headache specialist (Prof. M.T.), who was the senior investigator in this research.

The migraine subjects were then instructed to fill out a headache diary during the month ahead (Razeghi Jahromi et al., 2018). A headache diary records the number of headache days, the duration, and severity of each headache attack, and the number of times abortive drugs were used. Participants were contacted by telephone weekly during the month. On their second visit after 1 month, at the time the headache diaries were collected, blood samples were taken from the CM and EM subjects in the ictal phase (*n* = 63) and EM subjects in the interictal phase (*n* = 37) (72 h after most recent attack). In the case of ictal phase, patients were instructed not to take their usual painkillers or specific migraine medication until blood samples were taken.

The mean severity of the headache was measured by using the visual analog scale (VAS), which rates pain intensity from 0 (almost no pain) to 10 (worst possible pain experienced by a person).

### 2.3 Serum prolactin assessment

The blood samples (10 mL) were drawn from the antecubital vein of all participants on above mentioned dates. At the time of sampling, participants were asked if they are currently experiencing a headache or not. All serum samples were sent to the biochemistry laboratory of Sina Hospital, Tehran, Iran. Serum concentrations of prolactin were measured using enzyme‐linked immunosorbent assay (ELISA) kits from Shanghai Crystal Day Biotech (China).

### 2.4 Sample size and statistical analysis

An a priori sample size was not calculated; thus, this sample size was of convenience. A Shapiro‐Wilk test was used to test the normal distribution of the data. The chi‐square test was performed for comparison of categorical variables. The data were expressed as mean (± standard deviation) for parametric variables. In the case of normally distributed continuous data, the analysis of variance (ANOVA) test was performed to compare the mean prolactin levels, headache characteristics, age and BMI among the migraine groups and the control group. The Spearman's correlation test also was used to estimate the correlations between the serum concentration of serum prolactin and the number of headache days as well as the BMI of the subjects. Analysis of covariance (ANCOVA) was used to eliminate the effect of BMI as a confounder. Statistical significance levels were considered to be *p* < .05 (one‐tailed test) in all analyses. The analyses were conducted using SPSS 21(IBM Armonk, NW, USA).

## 3 RESULTS

### 3.1 Basic characteristics of migraine subjects and control group

The basic characteristics of the subjects are summarized in Table 1. No significant differences were observed in the distributions for age, sex between groups. From 30 subjects in the control group with mean ± standard deviation (SD) age of 41 ± 8, 73.3% were women. 78.4% of the interictal EM, 87.3% of the ictal EM subjects, and 74.4% of the chronic migraine patients were female. The mean age of the chronic migraine, ictal EM, and interictal EM were 39 ± 8, 38 ± 8, and 37 ± 12, respectively.

**TABLE 1:** Baseline characteristics of the studied participants

		Control (*n* = 3 0)	Interictal EM (*n* = 37)	Ictal EM (*n* = 63)	CM (*n* = 39)	*p* Value
Age (year)^a^		41 ± 8	37 ± 12	38 ± 8	39 ± 8	.483*
BMI (kg/m^2^)^a^		24.88 ± 3.70	24.24 ± 4.36	26.29 ± 3.91	26.50 ± 4.38	.038*
Sex^b^	Female	22 (73.3%)	29 (78.4%)	55 (87.3%)	29 (74.4%)	.290**
	Male	8 (26.7%)	8 (21.6%)	8 (12.7%)	10 (25.6%)	

^a^ Mean ± SD.

^b^ Frequency (%).

* *p* Values based on one‐way ANOVA test.

** *p* Values based on chi‐square test.

EM, episodic migraine; CM, chronic migraine.

Regarding BMI, significant differences were found between groups (*p* = .038).

### 3.2 Headache characteristics

Table 2 shows the headache characteristics of the three migraine groups. The severity of headache according to VAS criteria was significantly higher in CM than in EM subjects. There were no significant differences in the duration of attacks between groups.

**TABLE 2:** Headache characteristics and analgesic consumption between groups

	CM	Ictal EM (*n* = 63)	Interictal EM (*n* = 37)	*p* Value*
Number of headache days	23.89 ± 7.24^a,b^	8.45 ± 3.32^a^	7.43 ± 3.24^b^	<.001
Attack duration (h)	22.46 ± 12.89	22.63 ± 15.07	21.75 ± 11.56	.951
Headache severity (VAS)	12.33 ± 14.09^a,b^	7.55 ± 1.77^a^	7.25 ± 2.16^b^	.004
Number of analgesic medications	13.25 ± 10.28^a,b^	5.76 ± 4.57^a^	5.47 ± 3.48^b^	<.001

All values are mean ± SD. *p* Value < .05 was considered significant.

* One‐way ANOVA test was used to compare between groups.

^a,b^ *p* Values < .05.

EM, episodic migraine; CM, chronic migraine.

### 3.3 Serum prolactin levels in studied groups

**FIGURE 1:**
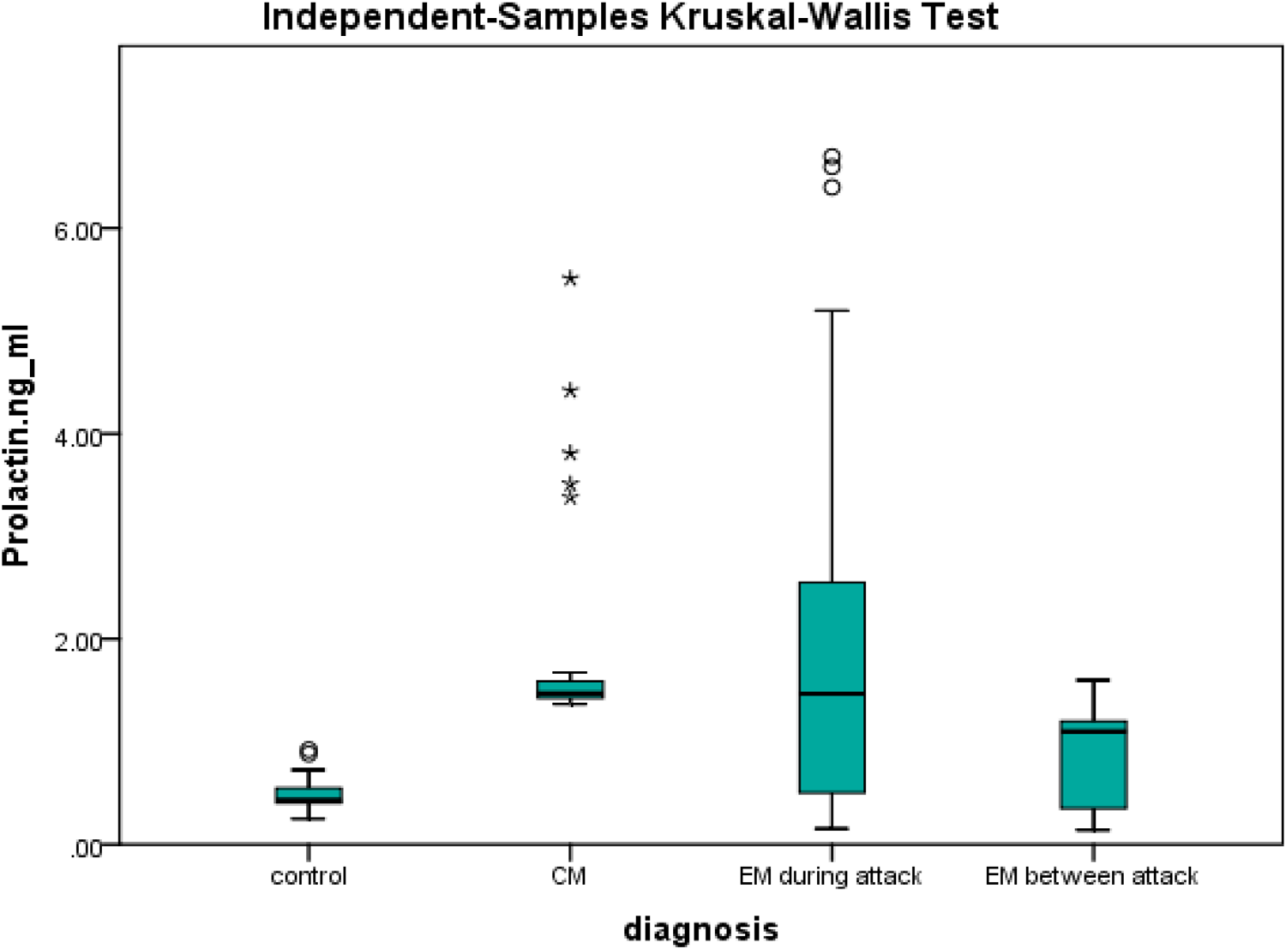
Serum prolactin levels for migraineurs and control groups. EM: episodic migraine; CM: chronic migraine.

**FIGURE 2:**
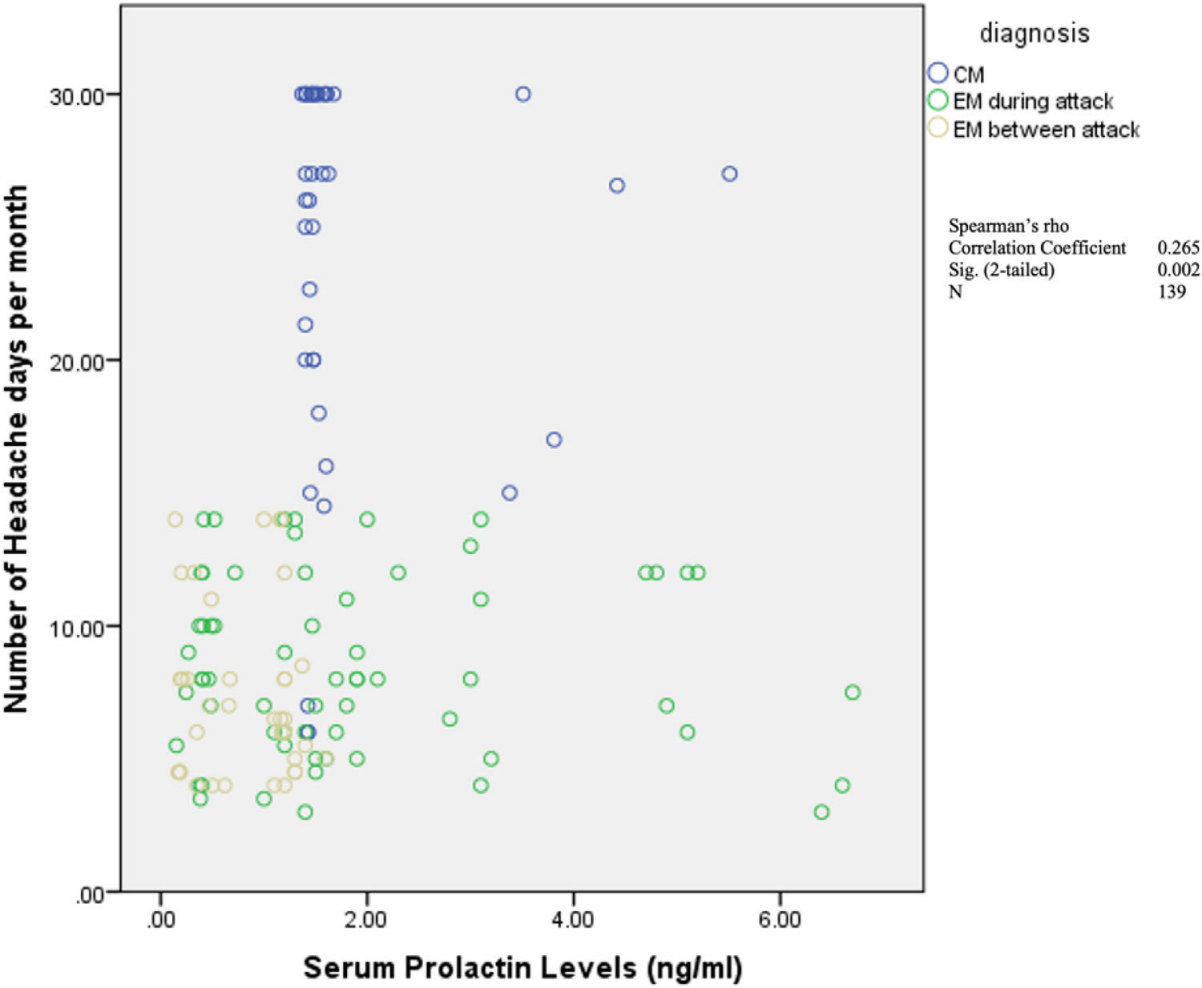
The correlation between prolactin concentration and the mean number of headache days per month. EM: episodic migraine; CM: chronic migraine.

The serum levels of prolactin in the four groups are shown in Table 3 and Figure 1. A significant difference was observed between the control, CM, ictal EM, and interictal EM subjects. The mean ± standard deviation (SD) of serum prolactin levels of the CM subjects (1.82 ± 0.94) and ictal EM subjects (1.93 ± 1.70) were comparable and significantly higher than for interictal EM subjects (0.82 ± 0.46) and control subjects (0.49 ± 0.15) (*p* < .001). Although the mean serum concentration of prolactin among the interictal EM subjects tended to be higher than for the control subjects, this difference was not statistically significant. Moreover, after considering BMI as a confounder in ANCOVA model, serum prolactin levels remained significant in the mentioned groups (*p* < .001). In Table 1, it was shown that BMI has a significant difference between the groups. Since BMI can affect both prolactin levels and migraine headache (D'Souza & Siat, 2019; O'Fallon et al., 2021; Rahardjo et al., 2022), it was considered as a confounding factor. Additionally, the Spearman's correlation test showed significant correlations between serum prolactin levels and the number of headache days for migraineurs (Figure 2).

**TABLE 3:** Serum prolactin levels by group

	Interictal EM	Ictal EM	CM	Control	*p* Value*	*p* Value**
Prolactin level (ng/mL)	0.82 ± 0.46^c,d^	1.93 ± 1.70^b,c^	1.82 ± 0.94^a,d^	0.49 ± 0.15^a,b^	<.001	<.001

All values are mean ± SD.

* Obtained from the ANOVA test for comparison of data among the groups.

** Obtained from ANCOVA test adjusted for the BMI among the groups.

^a,b,c,d^ *p* Values < .05.

EM, episodic migraine; CM, chronic migraine.

## 4 DISCUSSION

In the current study, we compared the level of prolactin in migraineurs with age‐ and sex‐matched headache‐free subjects and investigated its association with the number of headache days. It was shown that prolactin levels in subjects with ictal EM did not differ significantly from those with CM. A major finding was that both CM and ictal EM subjects had significantly higher prolactin levels than the interictal EM and control subjects. It was also found that interictal EM subjects out of the acute phase had higher levels of prolactin than the headache‐free subjects, but the difference between groups was not significant.

Significant correlations were found between the serum prolactin levels and the number of headache days among migraineurs (*p* = .002).

Although studies evaluating prolactin levels in subjects with migraines have shown different results, taken as a whole, our results on the levels of prolactin in migraineurs compared to controls appear to be corroborated by previous evidence in this field (Cavestro et al., 2006; Li et al., 2018; Nattero et al., 1986; Taha & Mohammed, 2019; Vega et al., 2017). Li et al. (2018) assessed serum different hormones related to female gonadal function including prolactin of migraineurs and analyzed the relationship between these hormone levels and migraine characteristics. The serum levels of these hormones were compared between 119 migraineurs, 42 subjects with tension‐type headaches, and 30 headache‐free control subjects. They concluded that the levels of prolactin were significantly higher in migraineurs than the control group.

The findings of the present study showed a lack of significant differences between the interictal EM group and control group. Guldiken et al. (2011) investigated the relationship between prolactin levels and atherosclerosis risk factors of soluble CD40 ligand (sCD40L) and high‐sensitivity C‐reactive protein (hs‐CRP) in migraineurs between attacks. In accordance with our findings, they found no a significant difference between the prolactin levels of migraine subjects in their interictal phase and the controls.

The outcomes of some studies which reported that no significant difference was found between prolactin levels in migraineurs compared to the control group were not consistent with those of the present study (Guldiken et al., 2011; Seddighi & Dehghani, 2002). Seddighi and Dehghani (2002) gauged the relationship between acute migraine attacks and changes in prolactin in 20 subjects with migraine and 20 subjects with nonmigraine headache. As opposed to our study, they concluded that serum prolactin was not elevated in the acute phase of migraine. In contrast to the present study, Masoud and Fakharian (2005) who measured serum prolactin levels during acute attacks in 37 subjects with migraine and 37 with nonmigraine headaches reported that prolactin levels decreased during the acute migraine headaches. One of the reasons for the difference in conclusions in these studies compared to the present study could be their small sample size. Another reason could be the lack of separate examinations of prolactin levels in subjects with EM and CM.

It is worth mentioning that most previous studies only evaluated the difference of prolactin level among migraineurs and a headache‐free control group (Cavestro et al., 2006; Guldiken et al., 2011; Li et al., 2018; Masoud & Fakharian, 2005; Seddighi & Dehghani, 2002; Taha & Mohammed, 2019; Vega et al., 2017), while in our study, we compared prolactin levels in patients with chronic migraine, episodic migraine in ictal and interictal phases and control subjects separately. In addition to, the correlation between prolactin levels and the number of headache days was assessed in our study. Our findings showed a significant correlation between the number of headache days and serum prolactin levels among EM and CM groups. It could be hypothesized that alterations in prolactin levels may contribute to the activation of headaches in migraineurs and increase the number of headache days that may predispose a person to chronic migraine (Xu et al., 2020)

Given the variation in prolactin levels in serum during headache attacks, previous studies have suggested that prolactin may be involved in the mechanism of the attacks (Hering & Kuritzky, 1992; Horrobin, 1973). In addition, hyperprolactinemia in pituitary tumor in which patients often have headaches supports the idea that increased levels of prolactin has been linked to increased migraine attacks (Bosco et al., 2008). In patients with a pituitary tumor, dopaminergic agonists, including cabergoline and bromocriptine, can normalize prolactin levels and decrease headache attacks (Pineyro et al., 2017). The mechanism through which prolactin could affect headache attacks is unknown. Among the neuroendocrinological alterations, hypothalamic‐pituitary axis disorders are believed to be engaged in headache pathogenesis (Cavestro et al., 2006). There are several possible reasons for a change in prolactin levels during migraines, including serotonin hyperfunction as a contributor to dopaminergic dysfunction, hypersensitivity of dopamine receptors based on the observation that migraineurs showed higher prolactin after taking dopaminergic antagonists and the reduced responsiveness of pituitary lactotroph cells to the action of dopaminergic agents (Cavestro et al., 2006).

As mentioned, prolactin is able to modulate central and peripheral neurons. It has stimulating effects on the immune system and nociceptive neurons. The transient receptor potential vanilloid1 (TRPV1) channel, a nociceptive channel, is important in pain mechanisms. It is also sensitized by prolactin in trigeminal sensory neurons, where the activation of TRPV1 is mediated by protein kinase C (PKC) and phosphatidylinositol 3‐kinase (PI3K). This pathway potentiates capsaicin‐evoked currents, calcium influx, and αCGRP release, thus affecting the nociceptors and increasing migraine pain. It should be noted that PKC and PI3K are involved in regulating TRPV1 channel activity through some inflammatory mediators (Artero‐Morales & González‐Rodríguez, 2018; Chen et al., 2020; Patil et al., 2013).

Despite these, even normal levels of prolactin could contribute to the sensitization of trigeminal sensory neurons and migraine attacks (Chen et al., 2020). However, it is difficult to understand the entire process because of the broad relationships and interaction between all hormones and mediators.

One of the strengths of the present study that sets it apart from other studies is that we evaluated the level of prolactin in the ictal and interictal periods in the EM group and compared the values with those of the CM group as well as with headache‐free controls. Further, in this study, EM and CM were accurately diagnosed by an expert headache specialist‐neurologist and the results were compared with age‐ and sex‐matched headache‐free controls.

Some of the limitations of this study include the case‐control design, which could be a source of bias when interpreting the findings because a cause‐and‐effect relationship cannot be confirmed between migraine and prolactin. In addition, various confounding factors such as stress, which can affect serum prolactin levels, were not considered in our study (Chen et al., 2020). Because of the inconsistency of prolactin levels in migraineurs, we suggest further investigation of hormone levels such as dopamine, which is a factor in migraine mechanisms and affects prolactin levels, as well as corticosterone levels, which could provide information on patients' stress levels.

## 5 CONCLUSION

In the present study, subjects with chronic and episodic migraine during the ictal phase were shown to have higher serum levels of prolactin than the episodic migraineurs in the interictal phase and age‐ and sex‐matched headache‐free individuals. These findings further support the hypothesis that there might be an association between increased prolactin concentrations and the induction of a migraine attack and this may increase the risk for transformation of EM to CM. To determine a possible causal relationship between the prolactin level and pathogenesis and development of migraine headaches or the progression of episodic migraines to chronic migraines, more studies with larger sample sizes should be undertaken.

## AUTHOR CONTRIBUTIONS

Study concept and design, patient selection, supervising all steps of the study, and editing the manuscript: M.T. Analysis and interpretation of data and editing the manuscript: Z.G. Drafting of the manuscript: S.H. Patient data acquisition: P.R and S.H. All the authors have read and approved the final version of the manuscript.

## CONFLICT OF INTEREST STATEMENT

The authors declare that they have no conflicts of interest.

### PEER REVIEW

The peer review history for this article is available at https://publons.com/publon/10.1002/brb3.3063.

## Data Availability

The datasets used and/or analyzed during this study are available from the corresponding author on reasonable request.
